# NLRP3 Inflammasome Activation Regulates Aged RBC Clearance

**DOI:** 10.1007/s10753-018-0784-9

**Published:** 2018-04-21

**Authors:** Li Qin, Zhao Fengyong, Zhang Jiamin, Yang Qixiu, Lu Geming, Xia Rongwei, Zhu Ziyan

**Affiliations:** 1 Blood Group Reference Laboratory, Shanghai Blood Center, Hongqiao Road 1191, Shanghai, 200051 China; 2 School of Life Science, East China Normal University, Shanghai, China; 3 Diabetes, Obesity and Metabolism Institute, Division of Endocrinology, Diabetes and Bone Diseases, the Mindich Child Health and Development Institute, Icahn School of Medicine at Mount Sinai, New York, USA; 4 Yunnan Qujing Central Blood Station, Qujing, Yunnan Province China

**Keywords:** NLRP3 inflammasome, aged erythrocytes, cytokines, THP-1

## Abstract

The NLR family pyrin domain-containing protein 3 (NLRP3) inflammasome is triggered by various stimuli. Whether the NLRP3 inflammasome is activated during the monocyte clearing of aged or damaged erythrocytes is unknown. This work aimed to determine whether the NLRP3 inflammasome is activated during the THP-1 cell engulfing of aged erythrocytes. In the study, THP-1 cells were treated with PMA and then coincubated with untreated red blood cells (RBCs), 42 °C-treated RBCs, immunoglobulin G (IgG) anti-D-sensitized RBCs, Rhnull/Rhmod RBC sample, hemoglobin, and RBC ghost. The activation of the NLRP3 inflammasome and production of some proinflammatory cytokines were determined using immunoblotting, cytometric bead array, and digital PCR. An NLRP3 inflammasome inhibitor was also used to evaluate the alteration of the NLRP3 activation and RBC clearance rate. The untreated RBCs, 42 °C-incubated RBCs, IgG-opsonized RBCs, Rhnull/Rhmod RBCs, RBC ghosts, and hemoglobin induced the THP-1-cell-mediated activation of the NLRP3 inflammasome and the production of inflammatory cytokines. The RBC clearance rate exhibited a positive correlation with the expression of proinflammatory cytokines. The NLRP3 inflammasome inhibitor reduced the NLRP3 activation and RBC phagocytosis rate. The NLRP3 inflammasome was activated during the clearance of the aged erythrocytes through unopsonized and opsonized pathways. However, the mechanism of such phenomenon needs to be further elucidated. Such mechanism may provide new insight into the assessment of the safety of transfusing long-storage RBC based on cytokine levels.

**Electronic supplementary material:**

The online version of this article (10.1007/s10753-018-0784-9) contains supplementary material, which is available to authorized users.

## INTRODUCTION

Red blood cells (RBCs) are senescent during *in vivo* circulation or *in vitro* storage. Hod and Spitalnik found that transfusing long-storage RBCs can cause multiple complications, such as sepsis, pneumonia, and multi-organ failure [1]. They hypothesized that the iron derived from damaged RBCs may play a key role in such inflammatory response.

Two different mechanisms of senescent erythrocyte clearance have been described in the phagocytosis of aged RBCs by monocytes, such as macrophages, predominantly in the spleen and liver. These mechanisms are termed as unopsonized and opsonized phagocytoses, which are controlled by the CD47–signal regulatory protein α (SIRPα) signaling pathway and the immunoglobulin G (IgG)–Fc gamma receptors (FcγR) signaling pathway, respectively [2, 3].

CD47 is a 50 kDa plasma membrane protein with an extracellular immunoglobulin-like domain, five transmembrane domains, and a short cytoplasmic tail. It is expressed on many cell types, including RBCs [4]. CD47 on RBCs is recognized by SIRPα, which is the ligand of CD47 on macrophages [5]. The interaction between the CD47 of normal expression on RBCs and SIRPα on macrophages sends a negative signal to macrophages that protects the RBCs from phagocytosis. A decreased CD47 expression on senescent RBCs has been demonstrated [6]; such decrease is always accompanied by the release of cell membrane microparticles or vesicles from the RBCs [7, 8]. The low expression of CD47 on the RBCs subsequently reduces the “not eat” signal through the CD47–SIRPα pathway. As a result, such RBCs are cleared by monocytes [2, 9]. On the other hand, the aged RBCs form Band 3 clusters and subsequently bind to antibodies and/or complements [10]. The phagocytosis of such RBCs can be induced following the ligation of receptors, such as FcγR [11].

Alblas et al. found that soluble CD47 recombinant protein can induce rat macrophages to produce NO, which is a marker of the M1 macrophage, through the CD47–SIRPα–Janus kinase 2 (JAK2)–signal transducers and activators of transcription (STAT) pathway [12, 13]. Their findings suggested that the aged RBCs cleared by monocytes through the CD47–SIRPα signaling pathway may trigger macrophage polarization and secrete cytokines. Whether the IgG–FcγR signaling pathway causes inflammatory reaction is currently unsupported.

The inflammatory reaction is complex, and many proteins are involved in the process. The inflammasome is an important protein complex in immune response [14]. To date, many different inflammasomes have been described [15]. For example, the NLR family pyrin domain-containing protein 3 (NLRP3) inflammasome is among the best characterized inflammasome and is involved in controlling the activity of caspase-1 and the maturation and secretion of the proinflammatory cytokines interleukin (IL)-1β and IL-18 [16]. Such inflammasome may also drive pyroptosis or mediate unconventional protein secretion [17].

The NLRP3 inflammasome can be triggered by pathogen-associated molecular pattern molecules (PAMPs) and/or danger-associated molecular pattern molecules (DAMPs), such as pathogens, DNA, and ATP, and can cause several diseases [18–20]. Transfusion-related acute lung injury (TRALI) is believed to be related to the DAMPs [21]. However, whether aged RBCs are DAMPs that can activate the NLRP3 inflammasome has not been confirmed. The safety of the transfusion of long-storage RBCs (old RBCs) in the clinic also remains controversial. The designated demarcation points between fresh RBCs and old RBCs are different, namely, 14 [22] and 21 days, respectively [23]. Several published data suggested that giving patients long-storage RBCs may cause adverse events, such as infection and even death [23, 24]; another opinion suggested that old RBCs do not increase the risk of patients [22, 25].

In the present study, we aimed to determine whether the low CD47 expression or IgG-opsonized RBCs can induce a proinflammatory response by activating the NLRP3 inflammasome, whether hemoglobin is the key factor in such response, and whether the NLRP3 pathway can regulate the macrophages that engulf aged RBCs and estimate the safety of transfusion of old RBCs by using an *in vitro* cell test.

## MATERIALS AND METHODS

### Collection and Treatment of Blood Samples

Whole blood was collected from healthy donors at the Shanghai Blood Center. All procedures have been reviewed and approved by the Shanghai Blood Center Medical Ethical Committee.

The erythrocytes were washed three times with a physiological saline solution. Then, the RBCs were divided into three aliquots. One aliquot was incubated at 42 °C for 2 h to prepare senescent erythrocytes, whereas another was sensitized with the IgG anti-D antibody (Shenxing, Shanghai Hemo-Pharmaceutical & Biological Inc., Shanghai, China). The remaining aliquot was not subjected to further treatment and marked as the untreated RBCs.

A suspect Rhnull/Rhmod blood sample was collected at the Yunnan Qujing Central Blood Station.

The CD47 expression on the RBCs was detected using flow cytometry (FACScan, BD Biosciences) with anti-CD47 (sc-12730, Santa Cruz) and FITC-conjugated secondary antibodies (115-095-003, Jackson ImmunoResearch). The indirect antiglobulin test was then used to determine whether the IgG anti-D antibody bound to the anti-D-treated RBCs.

### RBC Ghost Preparation

RBC ghosts were prepared as described by Bütikofer and colleagues [26], with some modification. Briefly, fresh RBCs from a healthy donor were washed at least three times with 0.9% saline, and 1 vol of packed RBCs was treated with 40 vol of hypotonic solution (10 mmol/L Tris–HCl, pH 7.5). The mixture was incubated in ice for 30 min and then centrifuged at 20,000×*g* at 4 °C for 20 min. The pellets were repeatedly washed with the same buffer until white in color (white ghosts, total RBC membrane largely free of hemoglobin). The ghosts were stored at − 80 °C.

The CD47 expression on the ghosts was detected using immunoblotting. Briefly, the ghosts were lysed in a RIPA buffer (50 mM Tris–HCl, 0.15 M NaCl, 1% Na-deoxycholate, 1 mM EDTA, 1% Nonidet P-40, 1 mM PMSF, 0.7 μg/mL pepstatin, 5 μg/mL leupeptin, 2 μg/mL aprotinin, and 1 mM Na_3_O_4_V). The protein was resolved using sodium dodecyl sulfate–polyacrylamide gel electrophoresis and then blotted onto a polyvinylidene membrane. The CD47 expression was analyzed with anti-CD47 (sc-12730) and goat anti-mouse IgG–horseradish peroxidase (sc-2005, Santa Cruz Biotechnology). The glycophorin A (GPA) detected using anti-GPA (BRIC 256) was used as internal control.

### THP-1 Cell Culture and Stimulation

In the study, 9 × 10^5^ human monocytic leukemia (THP-1) cells (Cell Bank, Chinese Academy of Sciences, Shanghai) were cultured in each well of a 24-well cell culture plate containing a RPMI-1640 medium (Gibco) supplemented with 10 IU/mL penicillin, 10 μg/mL streptomycin, and 100 ng/mL phorbol-12-myristate-13-acetate (PMA, P1585, Sigma-Aldrich) and without fetal bovine serum. Then, the cells were incubated in 5% CO_2_ at 37 °C for 48 h.

The PMA-treated THP-1 cells were allocated to two groups, namely, the groups in the absence and presence of the NLRP3 inhibitor. To prepare the NLRP3 inhibitor group, the NLRP3 inhibitor (NBP2-30141, Novus) was initially prepared into a stock solution of 25 mg/mL in dimethyl sulfoxide and then adjusted to a working solution of 2.5 mg/mL by using sterilized PBS. The working solution was added to the PMA-treated THP-1 cells to reach the final concentration of 2 μg/mL. After 5 min, each well was added with the untreated RBCs, 42 °C-treated RBCs, IgG-opsonized RBCs, Rhnull/Rhmod RBCs, hemoglobin (36A16356, Worthington), RBC ghost, and nigericin (XPO424B1014, Sangon) at 1 × 10^5^ RBCs per well. The final concentrations of hemoglobin, RBC ghost, and nigericin were 30 μg/mL, 30 μg/mL, and 30 μM, respectively. The culture system was incubated in 5% CO_2_ at 37 °C for 30 min or 4 h.

### Phagocytosis Assay

RBC clearance was determined using a monocyte monolayer assay (MMA) and live cell imaging system.

The MMA was operated in accordance with the technique set by Olsson and colleagues [27], with some modification. Briefly, after incubation for 30 min, the THP-1 and RBC coincubated system was washed with PBS and then stained with a Wright–Giemsa dye. Phagocytosis was detected using phase-contrast light microscopy (IX51, Olympus, Japan) and quantified as phagocytic rate, determined by the number of THP-1 that engulfed the RBCs per 100 THP-1 cells. The experiment was repeated seven times.

The phagocytosis of the untreated RBCs, IgG-opsonized RBCs, and Rhnull/Rhmod RBCs was also tested using a live cell imaging system (DeltaVision, GE Healthcare Life Sciences). Subsequently, 1 × 10^4^ THP-1 cells were plated into 35-mm dishes and treated with PMA as described earlier for 48 h. The dish was placed in the imaging system, and then six microscopic fields were selected per dish. Afterward, 1 × 10^5^ RBCs were added to the dish and automatically continuously photographed for 30 min in 5% CO_2_ at 37 °C. The results were determined by the number of RBCs that were engulfed by one THP-1 per unit time.

### Cytokine Analysis by Using a Cytometric Bead Array (CBA) Kit

Cytokines, including IL-1β, IL-6, IL-12p70, interferon (IFN)-γ, and tumor necrosis factor (TNF)-α, were detected using the BD CBA kit (6049889) along with five beads in a series (6008540, 6120549, 6183997, 6315650, and 6071701). The tested culture supernatants were collected after 30 min or 4 h of incubation. The results were collected using flow cytometry (FACScan, BD Biosciences) and analyzed using FACPArray.

### Digital PCR (D-PCR)

After the THP-1 cells and RBCs were coincubated for 30 min, the total RNA from the THP-1 cells was extracted using an RNA kit (DP419, Tiangen, China). The concentrations of the RNA samples were determined using NanoDrop Microvolume Spectrophotometers and Fluorometers (Thermo Scientific). The samples were adjusted to the same concentration as they were reversed transcribed to cDNA by using the PrimeScript TM II Kit (6210A, Takara, Japan).

The mRNA expressions of NLRP3, IL-1β, Il-6, IL-12p40, IFN-γ, and β-actin were detected using D-PCR. The primer sequences are presented in Table 1. The D-PCR reactions were performed in a 20 μL solution containing 10 μL of ddPCR EvaGreen Supermix (Bio-Rad), 100 nM primers, and approximately 60 ng template cDNA. The PCR reaction system was mixed with 70 μL droplet generation oil (Bio-Rad) by using a QX100 Droplet Generator (Bio-Rad). The droplets (40 μL total volume) were then transferred to a 96-well plate and covered with a pierceable foil heat seal on a PX1 plate sealer (Bio-Rad). PCR amplification was performed at 37 °C for 10 min; then, 40 cycles of amplification was run at 95 °C for 30 s and 60 °C for 1 min, 1 cycle at 90 °C for 5 min, and finally at 4 °C. The samples were analyzed on a QX200 droplet reader (Bio-Rad). Final evaluation was implemented using the QuantaSoft Software version 1.2.10.0 (Bio-Rad).

**Table 1:** Used Primers

Primer name	Sequence
NLRP3 F	5′-GTGTTTCGAATCCCACTGTG-3′
NLRP3 R	5′-TCTGCTTCTCACGTACTTTCTG-3′
IL-1β F	5′-TAAAGCCCGCCTGACAGAA-3′
IL-1β R	5′-GGAGCGAATGACAGAGGGTTT-3′
IL-6 F	5′-CCAGGAGCCCAGCTATGAAC-3′
IL-6 R	5′-CCCAGGGAGAAGGCAACTG-3′
IL-12p40 F	5′-CGGTCATCTGCCGCAAA-3′
IL-12p40 R	5′-CCCATTCGCTCCAAGATGAG-3′
IFNγ F	5′-CCAACGCAAAGCAATACATGA-3′
IFNγ R	5′-TTTTCGCTTCCCTGTTTTAGCT-3′
β-Actin F	5′-CCTGGCACCCAGCACAAT-3′
β-Actin R	5′-GCCGATCCACACGGAGTACT-3′

### NLRP3 Inflammasome Activation Test

The collected THP-1 cells were lysed in a RIPA buffer. The expression of NLRP3 inflammasome was detected using immunoblotting with anti-NLRP3 (AG-20B-0014-C100, AdipoGen).

### Statistics

Data were presented as means ± SE. Statistical analyses were conducted using Student’s *t* test by SPSS 16.0 (Chicago, IL, USA). Statistical significance was considered at the 95% level (*p* < 0.05).

## RESULTS

### CD47 Expression on RBCs and Ghosts

**Fig. 1:**
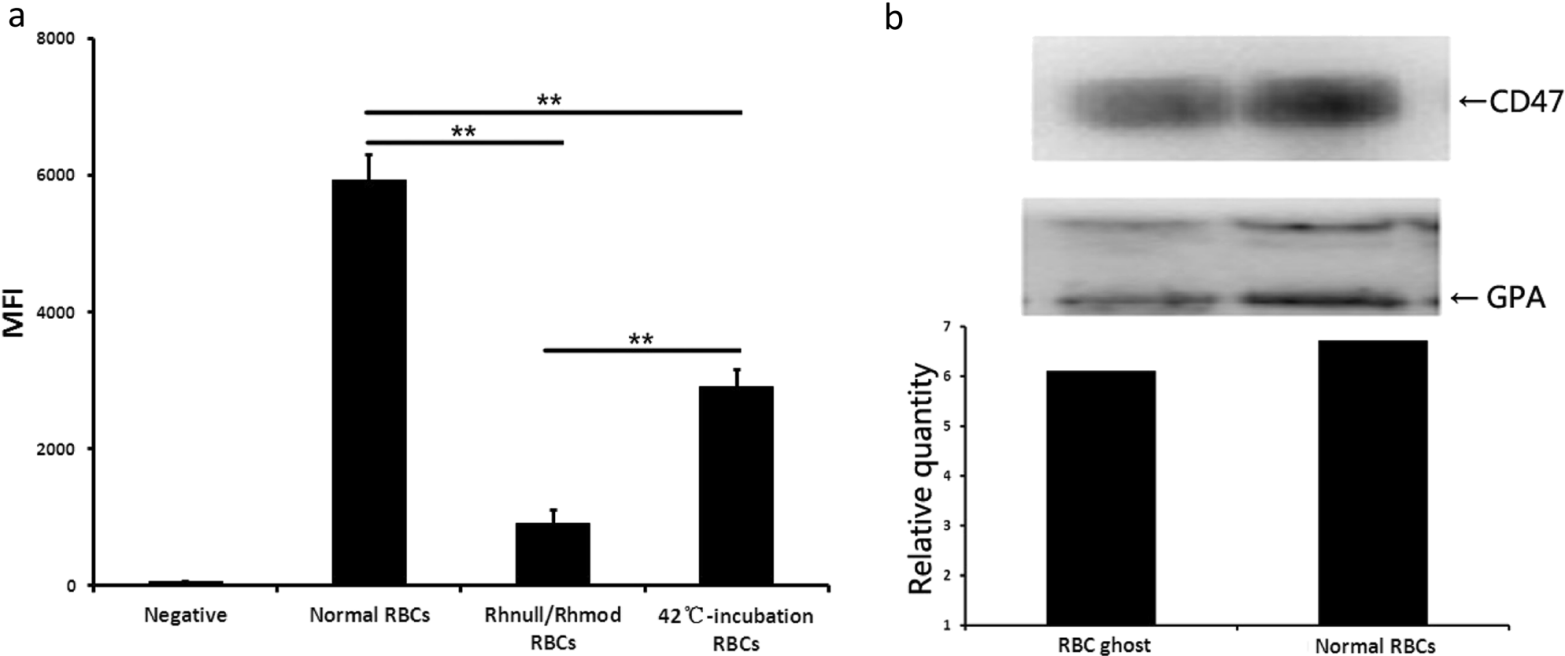
CD47 expression detected using flow cytometry and immunoblotting. **a** CD47 expression on normal RBCs (*n* = 7), Rhnull/Rhmod RBCs (*n* = 1, test in triplicate), and 42 °C-incubated RBCs (*n* = 3) tested using flow cytometry. RBCs without any CD47 expression were not reported in humans. The negative result was obtained by the normal RBCs that were reacted directly with the secondary antibodies. The MFI was used to show the CD47 levels. The MFIs of the negative result, normal RBCs, Rhnull/Rhmod RBCs, and 42 °C-incubated RBCs were 55 ± 7, 5938 ± 368, 912 ± 194, and 2909 ± 252, respectively. **b** CD47 expression on the RBC ghost and normal RBCs detected using immunoblotting. The relative amount obtained by the CD47 light density/GPA light density ratio was used to display the CD47 expression. The CD47 level on the RBC ghosts was similar to that on the normal RBCs. **p < 0.01.

The CD47 expression levels on the 42 °C-treated RBCs and Rhnull/Rhmod RBCs were determined using flow cytometry. Results were estimated by mean fluorescence intensity (MFI). The detailed results are shown in Fig. 1a.

The CD47 expression on the ghosts was analyzed using immunoblotting, and results are shown in Fig. 1b.

### Clearance Rate of the Different Treated RBCs

**Fig. 2:**
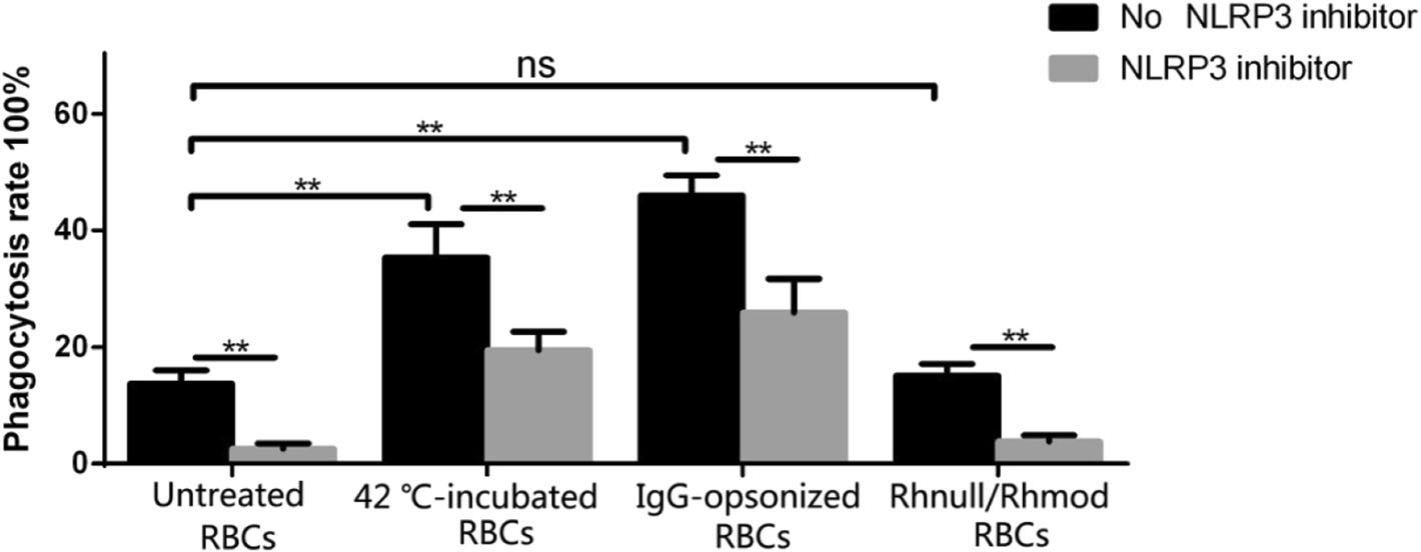
Phagocytosis rates of the different groups of RBCs. The MMA results showed that the phagocytic rate of IgG-opsonized RBCs, 42 °C-incubated RBCs, Rhnull/Rhmod RBCs, and untreated RBCs were 46.00% ± 3.46%, 35.32 ± 5.71%, 15.05% ± 2.07%, and 13.72% ± 2.29%, respectively. The IgG-opsonized RBCs exhibited the highest tendency for clearance by THP-1 cells, whereas the phagocytosis rate of the Rhnull/Rhmod RBCs was not significant compared with which of untreated RBCs. In the presence of the NLRP3 inhibitor in the culture system, the phagocytic rates of the RBCs were downregulated, and the phagocytic rate of the IgG-opsonized RBCs, 42 °C-incubated RBCs, Rhnull/Rhmod RBCs, and untreated RBCs became 25.94% ± 5.79%, 19.46 ± 3.18%, 3.78% ± 1.07%, and 2.54% ± 0.92%, respectively. **p < 0.01. *ns* not significant.

The phagocytosis rates of the different groups of RBCs are shown in Fig. 2. The phagocytosis images obtained from the phase-contrast light microscopy are displayed in the supplementary data (SFig. 1).

Whether the existence of the NLRP3 inhibitor is included or excluded, the IgG-opsonized RBCs exhibited the highest tendency for clearance by THP-1 cells, whereas the phagocytosis rate of the Rhnull/Rhmod RBCs was not significant compared with that of the untreated RBCs. The NLRP3 inhibitor downregulated the phagocytosis rate of all the kinds of RBCs.

The dynamic graphics of the THP-1 cells engulfing the untreated RBCs, IgG-opsonized RBCs, and Rhnull/Rhmod RBCs obtained using the live cell imaging system showed that the IgG-opsonized RBCs exhibited the highest tendency to be cleared. Two THP-1 cells in the selected microscopic fields pursued the IgG-opsonized RBCs actively, but the captured RBCs escaped. One untreated RBC was engulfed by a THP-1 cell, and no Rhnull/Rhmod RBC was found to be cleared by the THP-1 cells during the 30 min observation stage. In addition, most of the Rhnull/Rhmod RBCs became echinocytes during the 30-min test. The dynamic graphics are shown in the supplementary data.

### Activation of NLRP3

The activation of the NLRP3 inflammasome was examined using the CBA kit, D-PCR, and immunoblotting.

**Fig. 3:**
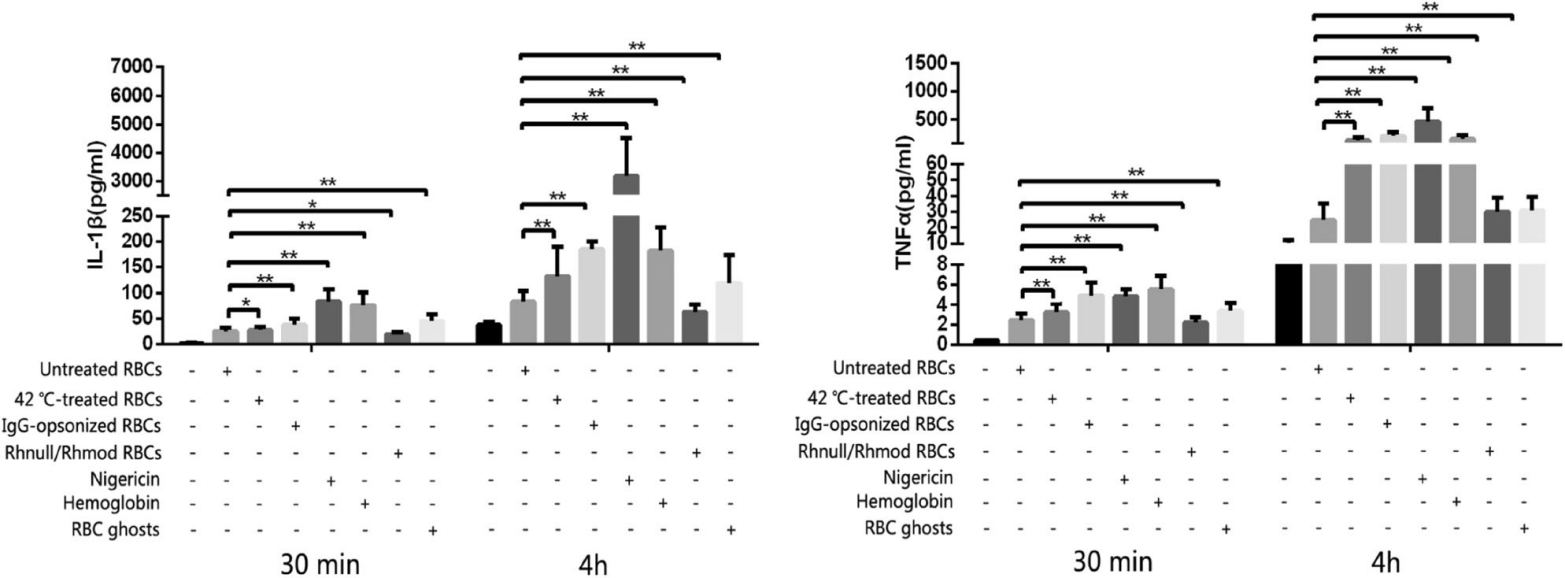
IL-1β and TNFα production detected using the CBA assay. The negative control was the THP-1 cells only. Without considering the reaction time, nigericin (the NLRP3 activation positive control) triggered the THP-1 to produce the most IL-1β. Hemoglobin was the strongest stimulus of TNFα expression in the 30 min incubation. When the reaction was prolonged to 4 h, the cytokine expression evidently increased, and the strongest irritant became nigericin. The Rhnull/Rhmod RBCs exhibited the poorest ability among the RBCs, even less to that of the untreated RBCs, in inducing THP-1 to produce IL-1β or TNFα. *p < 0.05; **p < 0.01.

**Fig. 4:**
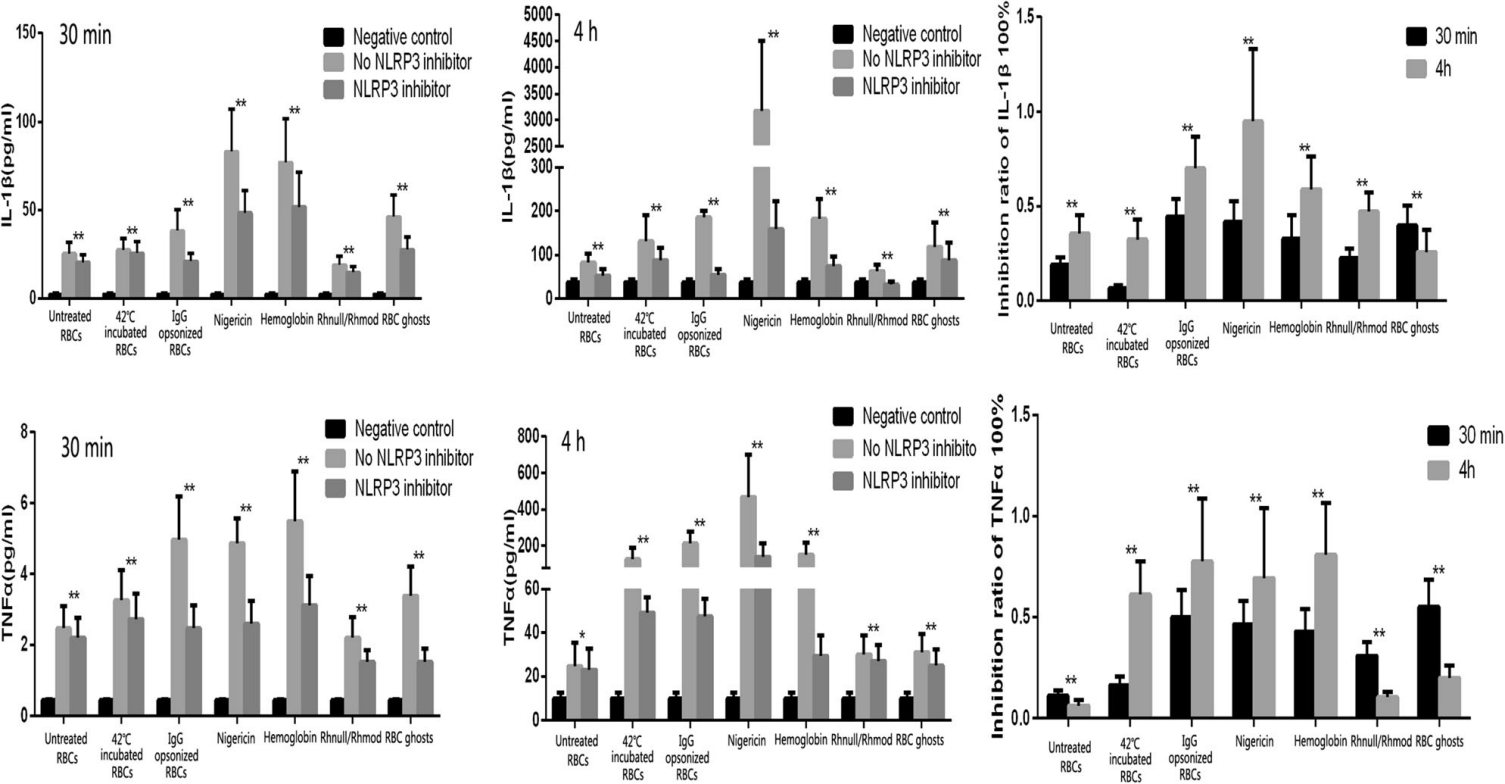
Effect of NLRP3 inhibitor detected using CBA assay. The negative control was the THP-1 cells only. The expression of IL-1β or TNFα showed a similar tendency in the 30-min and 4-h reactions. The NLRP3 inhibitor significantly downregulated the expression of the two proinflammatory cytokines in both the 30-min and 4-h reactions. The NLRP3 inhibitor showed a higher inhibition ratio in most groups at 4 h incubation than at 30 min incubation, except in the RBC ghost group in both IL-1β and TNFα productions and in the untreated RBC and Rhnull/Rhmod groups in the TNFα expression. The inhibition rate of the NLRP3 inhibitor was associated with the ability of the stimulus to induce the proinflammatory cytokine production. Nigericin, the IgG-opsonized RBCs, and hemoglobin were the three strongest stimulants to IL-1β secretion. After the 4-h reaction, the expression levels of IL-1β in the three groups were downregulated to 95.0, 70.0, and 59.1%, respectively. *p < 0.05; **p < 0.01.

The expression levels of IL-1β, IL-6, IL-12p70, IFN-γ, and TNF-α were detected using the CBA kit. In the 30 min incubation group, the expression levels of IL-1β and TNF-α were detected, but no signal of IL-6, IL-12p70, and IFN-γ was detected by the CBA system. When the incubation time was extended to 4 h, all the five cytokines were detected, but the FACPArray software suggested that the results of IL-6, IL-12p70, and IFN-γ were below the standard range and not meaningful. The results of IL-1β and TNF-α exhibited time dependence. The expression levels in the 4 h group were higher than those in the 30 min group (Fig. 3). The THP-1 cells could be induced to produce IL-1β and TNF-α production by the untreated RBCs, 42 °C-incubated RBCs, IgG-opsonized RBCs, Rhnull/Rhmod RBCs, RBC ghosts, and hemoglobin. The IgG-opsonized RBCs, hemoglobin, and RBC ghosts exhibited a strong ability to trigger the THP-1 to secrete IL-1β and TNF-α. When the NLRP3 inhibitor existed in the culture system, the cytokine expression was significantly downregulated regardless of the reaction. The inhibition rate of the NLRP3 inhibitor was associated with the reaction time and the ability of the stimulus to activate the NLRP3 inflammasome (Fig. 4).

**Fig. 5:**
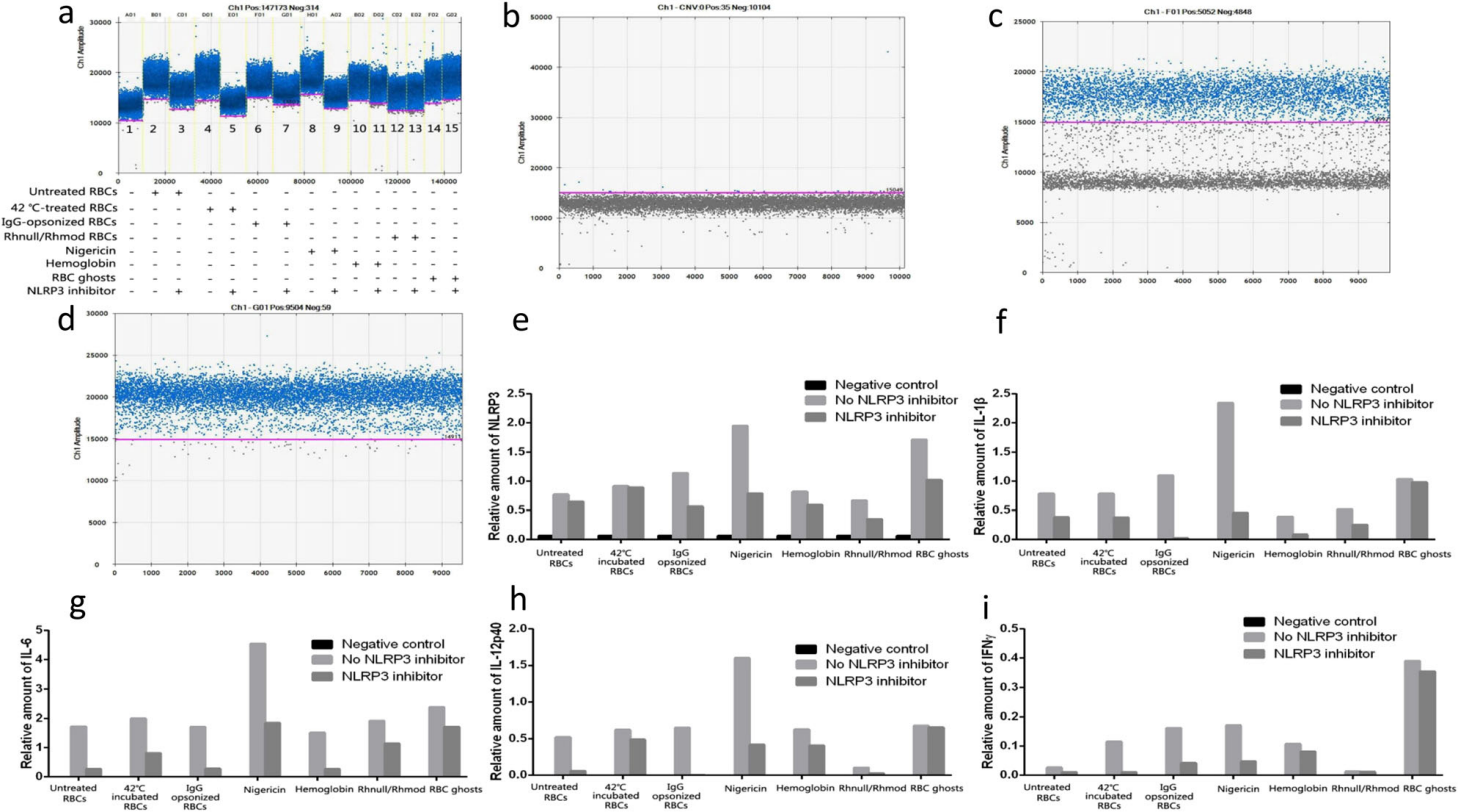
D-PCR results. **a** β-Actin concentrations of the 15 samples. Although the mRNA concentration of each sample was adjusted to the same value before the reverse-transcription PCR, the β-actin concentrations differed. The highest concentration was 8500 copies/μL (THP-1 and nigericin), and the lowest was 5980 copies/μL (THP-1; Rhnull/Rhmod RBCs). **b** NLRP3 results of the THP-1 cells only. Almost all the liquid drops showed negative results. **c** The NLRP3 results of the THP-1 cells and 42 °C-treated RBCs. The positive and negative findings were equal. **d** The NLRP3 results of the THP-1 cells and nigericin. Almost all the liquid drops showed positive results. **e**–**i** Relative amounts of NLRP3, IL-1β, IL-6, IL-12p40, and IFNγ. As the β-actin concentrations differed, the expression levels of NLRP3, IL-1β, IL-6, IL-12p40, and IFNγ were presented as relative amounts gained by dividing the target factor concentrations by the β-actin concentration. The obtained relative amounts were similar to those achieved using the CBA assay. The NLRP3 can be activated, and the mRNA levels of IL-1β, IL-6, IL-12p40, and IFNγ were upregulated by the untreated RBCs, 42 °C-incubated RBCs, IgG-opsonized RBCs, Rhnull/Rhmod RBCs, RBC ghosts, and hemoglobin. The IgG-opsonized RBCs, hemoglobin, and RBC ghosts strongly induced such reactions. When the NLRP3 inhibitor existed in the culture system, the mRNA levels of NLRP3 and cytokines were downregulated. QuantaSoft only showed one result for each detected factor; the *p* value could not be calculated.

The mRNA expression levels of NLRP3, IL-1β, IL-6, IL-12p40, and IFN-γ were determined using D-PCR. β-Actin was used as internal control. Although the mRNA concentration of each sample was adjusted to the same value before the reverse-transcription PCR, an evident difference was achieved among the results of β-actin under D-PCR (Fig. 5a). The expression levels of NLRP3, IL-1β, IL-6, IL-12p40, and IFN-γ were presented by relative quantity, which was the calculated ratio of the value of the target gene to internal control. The relative quantity of the detected gene achieved the same tendency to that determined by the CBA kit (Fig. 5e–i).

**Fig. 6:**
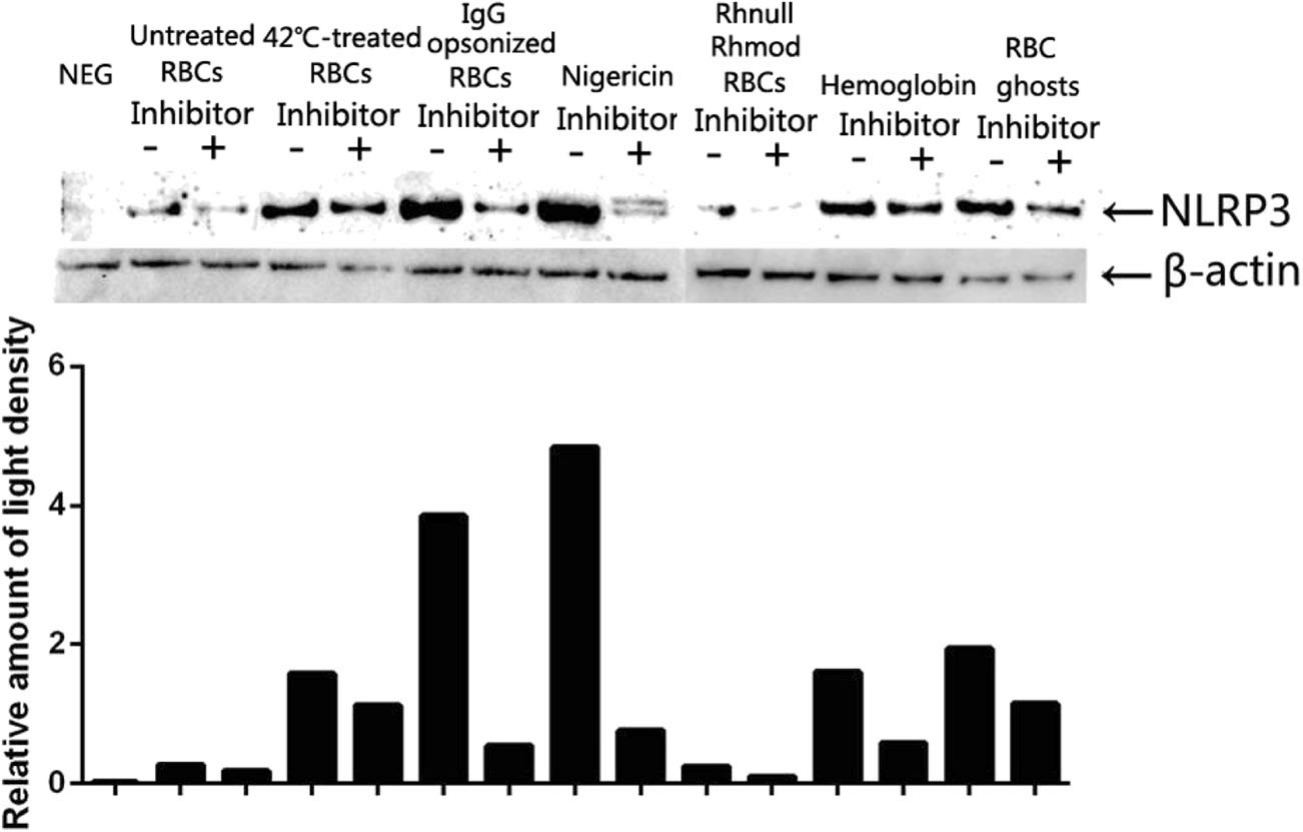
NLRP3 inflammasome activation detected using immunoblotting. NEG means negative control, only had THP-1 cells. Inhibitor: NLRP3 inhibitor. Results showed a similar tendency to that determined using the CBA kit and D-PCR. Thus, all the stimulants we used triggered the NLRP3 activation. Among these, nigericin, the IgG-opsonized RBCs, and the RBC ghosts exhibited a strong induction effect. The NLRP3 inhibitor significantly downregulated the NLRP3 activation.

The immunoblotting results of NLRP3 also showed a similar tendency to that determined by the CBA kit and D-PCR (Fig. 6).

In addition, the expression of NLRP3, IL-1β, IL-6, IL-12p70, IFN-γ, and TNF-α demonstrated a positive correlation with the RBC clearance rate; thus, the THP-1 exhibited a strong tendency to engulf the RBCs, and the more NLRP3 inflammasomes were activated, the more inflammatory cytokines, such as IL-1β and TNF-α, were secreted.

## DISCUSSION

CD47 is present abundantly on RBCs. It is the ligand of SIRPα expressed on monocytes. When CD47 binds with SIRPα, the monocytes receive a not eat signal, to protect the RBCs from clearance [2, 9]. The expression of CD47 on aged RBCs sharply decreases. The lost or low expression of CD47 then downregulates the phagocytosis-inhibiting signal and results in the clearance of aged RBCs. We used 42 °C treatments to simulate the low CD47 expression on RBCs. Overheating may induce blood cells to release vesicles [7]. Subsequently, part of the CD47 molecules on the RBCs transfer to vesicles. Finally, the CD47 expression on the RBCs is downregulated [8]. Our laboratory induced the aged RBCs through 37 °C incubation *in vitro* [28]. In this work, we increased the incubation temperature to 42 °C to accelerate the aging of the RBCs. The flow cytometry results showed that the CD47 expression on the 42 °C-incubated RBCs was approximately 51% of that on the untreated RBCs (Fig. 1a). Kriebardis and colleagues’ results showed that the CD47 on long-storage RBCs (more than 35 days at 4 °C) was less than 60% that of their fresh conditions [8]. The 42 °C treatment induced the old RBCs in a short time in terms of the amount of CD47.

The senescent RBCs in circulation are opsonized with autoantibodies and recognized by macrophage Fcγ receptors with the synergistic reaction of the complement system [9–11]. In the current study, we used anti-D IgG-opsonized RBCs to simulate the autoantibody-opsonized RBCs.

In addition, Rhnull/Rhmod, currently known as lowest CD47 expression human RBCs, was added to the culture system. Rhnull and Rhmod are two variants in Rh blood system and lack all the Rh blood antigens [29, 30]. These two kinds of blood types showed the same or similar results in the serological examination; the Rh phenotype should be determined at the gene level. The sample we used was not sequenced. Hence, the accuracy of the Rh phenotype was not determined. Hemoglobin and RBC ghosts were also added to the culture system. Nigericin was used to establish a NLRP3-positive control.

The results obtained from the CBA kit, D-PCR, and immunoblotting all suggested that the untreated RBCs, 42 °C-incubated RBCs, IgG-opsonized RBCs, Rhnull/Rhmod RBCs, RBC ghosts, and hemoglobin induced the THP-1 cell activation of the NLRP3 inflammasome and produced inflammatory cytokines. Among these stimulants, the IgG-opsonized RBCs, RBC ghosts, and hemoglobin strongly triggered the THP-1 cells to secrete the inflammatory cytokines, whereas the untreated RBCs and Rhnull/Rhmod RBCs only induced the THP-1 cells to produce a few cytokines.

The RBC clearance rate was closely related to the cytokine expression we detected. When the THP-1 cell showed a strong tendency to engulf the RBCs, more NLRP3 inflammasomes were activated and more inflammatory cytokines were secreted. Conversely, the NLRP3 inhibitor regulated THP-1 cells to uptake the RBCs. In the presence of the NLRP3 inhibitor, the ability of the THP-1 cells to engulf the RBCs was downregulated. The production of the inflammatory cytokines was also impaired.

Interestingly, the CD47 expression on the Rhnull/Rhmod RBCs was lower than that on the 42 °C-incubated RBCs (Fig. 1a). Considering the CD47–SIRPα mechanism, more Rhnull/Rhmod RBCs than 42 °C-incubated RBCs should be cleared by the THP-1 cells. However, our findings showed that the phagocytosis rate of the Rhnull/Rhmod RBCs was much lower than that of the 42 °C-incubated RBCs. This result was similar to that of Arndt and Garratty’s findings [31]. This phenomena suggested the existence of another mechanism to prevent the low CD47 expression on the Rhnull/Rhmod RBCs that were phagocytosed by the monocytes. In our study, the low clearance rate of the Rhnull/Rhmod RBCs decreased the secretion of the proinflammatory cytokines.

Furthermore, the CD47 amounts expressed on the RBC ghosts were similar to those on the untreated normal RBCs (Fig. 1b). We estimated that if the RBC ghosts can trigger the THP-1 cells to produce the proinflammatory cytokines, the cytokine amounts might be similar to those secreted by the untreated RBC-stimulated group. However, results showed that RBC ghosts exhibited a stronger ability to induce the NLRP3 inflammasome activation and produce proinflammatory cytokines than the untreated normal RBCs. These results suggested that the THP-1 cells possibly involved some other mechanisms besides the CD47–SIRPα signaling pathway in handling the stimulation from the RBC ghosts. Through our *in vitro* tests, we found that the NLRP3 inflammasome can be activated during the clearance of the aged erythrocytes through the unopsonized and opsonized pathways. The opsonized pathway can induce the THP-1 to secrete more proinflammatory cytokines than that produced by the unopsonized pathway. The hemoglobin exhibited a strong ability to trigger the NLRP3 activation. Thus, we speculated that when the RBCs were engulfed into the THP-1 cells whether through the CD47–SIRPα signaling pathway or through the IgG–FcγR signaling pathway, the hemoglobin in the RBCs may be a key factor in activating the NLRP3 inflammasome. The results of the THP-1 cocultured with the RBC ghosts suggested the presence of another protein or structure on/in the RBC that was involved in triggering the NLRP3 inflammasome activation, but the matter was not disclosed. The NLRP3 inhibitor downregulated the NLRP3 inflammasome activation during the clearance of the aged erythrocytes through the unopsonized and opsonized pathways. The phagocytosis rates of each RBC were reduced correspondingly.

In this work, several phenomena on the NLRP3 inflammasome activation and RBC clearance rate were observed. However, the mechanism of such phenomena was not thoroughly elucidated and requires further analysis. Nevertheless, this process remains as new candidate in explaining some transfusion side effects and offers a new insight into the evaluation of the safety of transfusing long-storage RBCs or reactions of blood group antigens with antibodies at the cytokine level. According to our results, fresh-RBC transfusion may have better effectiveness than old RBC transfusion. Some medicines may also be used in the future to downregulate the RBC clearance by adjusting some pathways, such as the NLRP3 pathway, to prolong the lifespan of the transfused RBCs post-transfusion.

## Electronic Supplementary Material

ESM 1 (DOCX 2532 kb)

ESM 2 (MOV 2837 kb)

ESM 3 (MP4 3785 kb)

ESM 4 (MOV 2805 kb)

## Abbreviations

NLRP3: NLR family pyrin domain-containing protein 3
RBC: Red blood cell
Ig: Immunoglobulin
SIRPα: Signal regulatory protein α
FcγR: Fc gamma receptors
JAK2: Janus kinase 2
STAT: Signal transducers and activators of transcription
IL: Interleukin
PAMPs: Pathogen-associated molecular pattern molecules
DAMPs: Danger-associated molecular pattern molecules
TRALI: Transfusion-related acute lung injury
IFN-*α*: Interferon *α*
TNF-γ: Tumor necrosis factor γ
MMA: Monocyte monolayer assay
PMA: Phorbol-12-myristate-13-acetate
