# Supplementary material for: NLRP3 Inflammasome Activation Regulates Aged RBC Clearance
Source: Inflammation. 2018 Apr 21;41(4):1361–71. doi: 10.1007/s10753-018-0784-9 (PMC6061012; doi:10.1007/s10753-018-0784-9)
Supplement: Supplementary file 1 — (DOCX 2532 kb) [file 10753_2018_784_MOESM1_ESM.docx]

**Supplementary data**


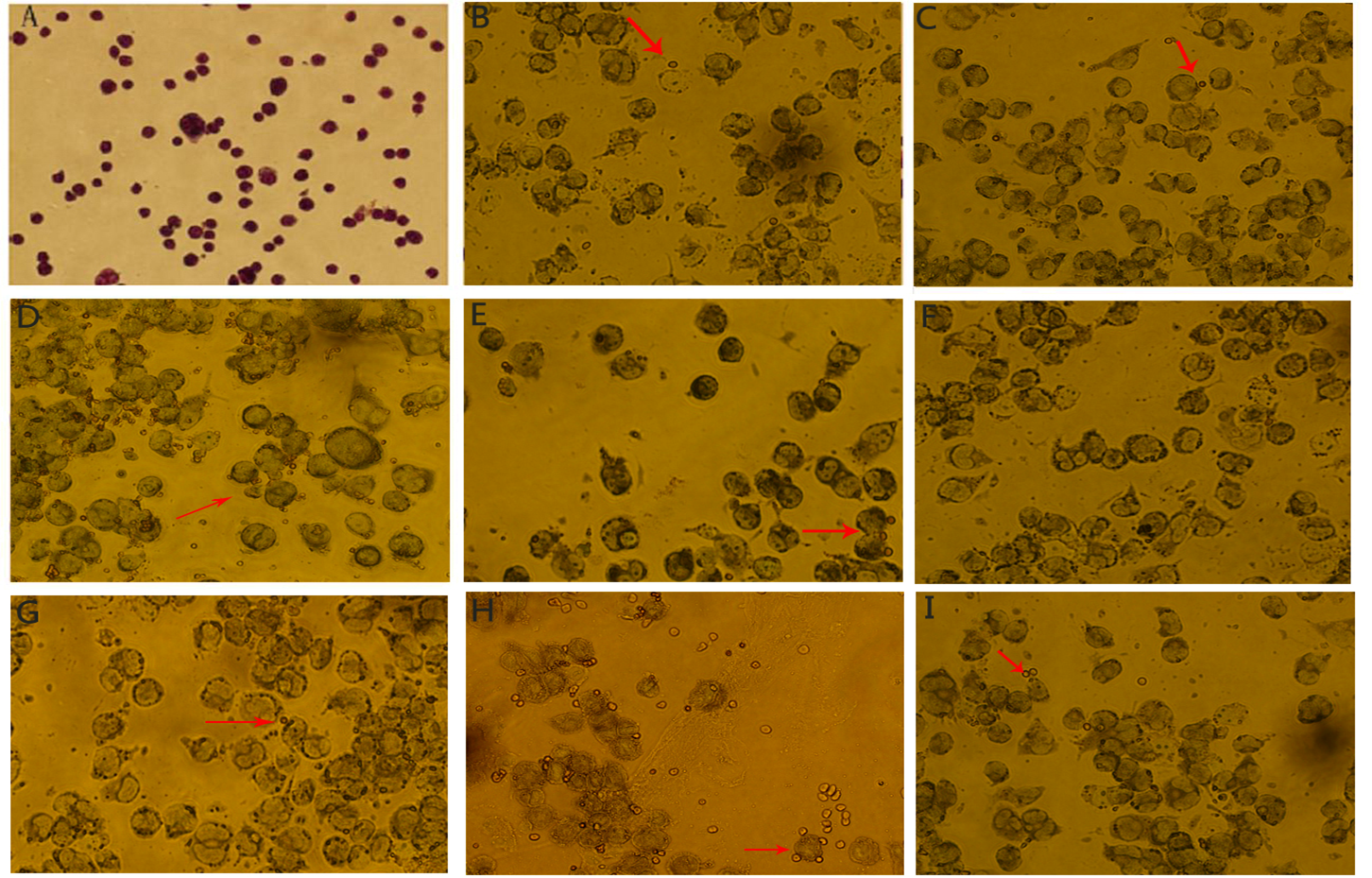


SFig. 1. THP-1 engulfed RBCs with different treatments. (A) Negative control, only THP-1 cells. (B) THP-1 and untreated RBCs. (C) Phagocytosis of 42 °C-treated RBCs. (D) THP-1-engulfed IgG-opsonized RBCs. (E) THP-1 and Rhnull/Rhmod RBCs. (F)–(I) NLRP3 inhibitor was added to the four culture systems. Besides the NLRP3 inhibitor, the culture mixtures were the same as those of (B)–(E). The large round cells are the THP-1 cells, and the small round cells are the RBCs. The arrow indicates the phagocytosis of RBC. The IgG-opsonized RBCs exhibited the highest tendency for clearance by THP-1 cells, whereas the phagocytosis rate of the Rhnull/Rhmod RBCs was not as high as expected. The NLRP3 inhibitor downregulated the phagocytosis rate of all the kinds of RBCs.

Legend for the dynamic graphics

The three dynamic graphics show the THP-1 cells engulfing the untreated RBCs, Rhnull/Rhmod RBCs, and IgG-opsonized RBCs, respectively. During the 30 min observation time, one untreated RBC was engulfed by one THP-1 cell. No Rhnull/Rhmod RBC was cleared by THP-1, but almost all the Rhnull/Rhmod RBCs became echinocytes during the 30 min test. Two THP-1 cells in the selected microscopic fields actively pursued IgG-opsonized RBCs by their pseudopodium, but the two captured RBCs escaped in the end. Results suggested that the IgG-opsonized RBCs exhibited the highest tendency to be cleared by THP-1 cells. Although the CD47 expression on the Rhnull/Rhmod RBCs was low, the phagocytosis rate of such RBCs was extremely low. Thus, a mechanism other than the CD47–SIRPα pathway may exist to protect the Rhnull/Rhmod RBCs from monocytic clearance.
